# Suicidal Ideation and Healthy Immigrant Effect in the Canadian Population: A Cross-Sectional Population Based Study

**DOI:** 10.3390/ijerph15050848

**Published:** 2018-04-25

**Authors:** Rasha Elamoshy, Cindy Feng

**Affiliations:** School of Public Health, University of Saskatchewan, Saskatoon, SK S7N 5E5, Canada; rasha.elamoshy@usask.ca

**Keywords:** suicidal ideation, immigrant, healthy immigrant effect, gender differences

## Abstract

Understanding suicidal ideation is crucial for preventing suicide. Although “healthy immigrant effect” is a phenomenon that has been well documented across a multitude of epidemiological and social studies—where immigrants are, on average, healthier than the native-born, little research has examined the presence of such effect on suicidal ideation. The objective of this study is to investigate if there is a differential effect of immigration identity on suicidal ideation and how the effect varies by socio-demographic characteristics in the Canadian population. Data from the Canadian Community Health Survey in year 2014 were used. Multivariate logistic regression was employed. Our findings indicated that recent immigrants (lived in Canada for 9 or less years) were significantly less likely to report suicidal ideation compared with non-immigrants. However, for established immigrants (10 years and above of living in Canada), the risk of suicidal ideation converged to Canadian-born population. Moreover, male immigrants were at significantly lower risk of having suicidal ideation than Canadian-born counterparts; whereas, female immigrants did not benefit from the “healthy immigrant effect”. Our findings suggest the need for targeted intervention strategies on suicidal ideation among established immigrants and female immigrants.

## 1. Introduction

Suicide is a worldwide public health concern which is responsible for close to million deaths per year [1,2]. In Canada, suicide is the ninth most common cause of death, with 4254 deaths reported in 2014 [3]. Non-fatal suicidal behavior represents a spectrum which may include suicidal ideations, suicidal plans, and non-fatal suicidal attempts. These behaviors have been linked to several negative consequences, including injury and hospitalization, and represent a burden on the health care system [2,4,5]. Suicidal ideation is a crucial component of the suicidal behavior, which often precedes suicidal attempts or completed suicide, and has been shown to be a strong predictor for suicidal death [6]. Therefore, understanding suicidal ideation is crucial for preventing the evolution to subsequent action [7,8]. A wide range of factors have been associated with suicidal ideation that involve mental, physical, social, and psychological characteristics [9]. These factors may include gender, age, lower socioeconomic status, psychiatric disorders, somatic symptoms, and physical illnesses [5,9,10]. 

Canada is a country primarily formed of immigrants and their descendants. Immigration is one of the main drivers for population growth and economic development in Canada. According to Canadian census data in 2016, 7.5 million foreign-born people came to Canada through the immigration process, and they represent more than 1 in 5 persons in Canada [11]. In 2036, it is projected to reach 30%, and both immigrants and second generations could represent one in two (44.2–49.7%) [12]. In this context, immigrants’ health represents a major component of the Canadian population health, and a key factor to be considered while planning future public health services and policies. Previous studies have shown that immigrants have better health profile than native-born counterparts, which is known as “the healthy immigrant effect”. This effect has been recognized in several countries, including the United States [13], Europe [14], Australia [15], and Canada [16]. In Canada, studies assessing physical health have shown that immigrants are protected against chronic illnesses (asthma, diabetes, and cancer) and disability or functional limitations [17]. Research on mental health aspects of Canadian immigrants also supported that the healthy immigrant effect extends to depression [18], anxiety [19], and other psychological conditions [17]. The most recognized explanation for immigrants’ health advantages is the selection process involved in migration, where those who have better health profile (physical and mental) are more likely to consider migration and to be accepted by the host country [20]. However, it has been also shown that these health advantages would attenuate by time [17,18,19]. This attenuation, despite not being fully understood, is usually linked to acculturation and adaptation to the host country environment [21,22].

The general understanding that the healthy immigrant effect is a widespread phenomenon experienced by all immigrants might be misleading. A growing body of evidence supports that immigrants’ health varied, not only by duration of stay in host country, but also across different age gender, ethnicity groups, source country, and health outcomes [17,23]. Therefore, research that examines these factors and how they influence immigrants’ health is necessary to better understand the full scope of the healthy immigrant effect. As such, the purpose of the current study is twofold: (a) to investigate whether “healthy immigrant effect” exists for suicidal ideation and (b) to assess whether such effect varies by socio-demographic factors among the Canadian population.

## 2. Materials and Methods

### 2.1. Survey

The survey data utilized in this study were based on the Canadian Community Health Survey (CCHS) data across Canada collected in the year of 2014 by Statistics Canada. It is an annual Canadian cross-sectional survey that gathers health-related information for the Canadian population. CCHS uses a multistage stratified cluster probability sampling and represents approximately 97% of the population over age 12 [24]. All estimates were weighted to approximate the distribution of demographic variables in the overall Canadian population.

### 2.2. Outcome Variable

The primary outcome for this study was lifetime suicidal ideation. In CCHS, lifetime suicidal ideations are assessed by asking “Have you ever seriously considered committing suicide or taking your own life?” The answer was either “yes” or “no”. This component was only asked for those respondents aged 15 and over at selected provinces in Canada: Newfoundland and Labrador, Prince Edward Island, New Brunswick, British Columbia, Nunavut. 

### 2.3. Primary Exposure Variable

The primary exposure variable was derived by combining the information on immigration identity and time since immigration. The immigration status was assessed as whether the participant is an immigrant or not. For immigrants, they were further asked about the length of time they spent in Canada since immigration, which was categorized into “0 to 9 years” to represent recent immigrants, and “10 or more years” to represent established immigrants. The primary exposure variable was then constructed as a categorical variable with three levels: non-immigrant, recent immigrant, and established immigrant.

### 2.4. Confounding Variables

Confounding variables included socio-demographic (age, gender, highest level of education attained, and household income) and clinical profile variables (chronic physical illness and mood disorder). Selection of these variables was based on existing scientific literature review [5,9,10].

Socio-demographic characteristics of participants included age, which was categorized into 4 groups: youth (<25 years), young adult (25–40 years), middle adult (40–65 years), and senior (>65 years); gender (male, female); level of education (less than secondary school graduation, secondary school graduation with no post-secondary education, some post-secondary education with no certificate/diploma or university degree, and post-secondary certificate/diploma or university degree); and household income level (<$20,000, $20,000 to <$39,999, $40,000 to <$59,999, $60,000 to <$79,999, ≥$80,000).

Chronic physical illness was a derived variable that combined responders who answered “yes” to at least one of the health conditions: asthma, diabetes, fibromyalgia, arthritis, migraine, chronic bronchitis, emphysema, chronic obstructive pulmonary disease, intestinal or stomach ulcers, stroke, bowel disorder, chronic fatigue, cancer, hypertension, and heart disease. Mood disorder was evaluated based on the question “Do you have a mood disorder such as depression, bipolar disorder, mania or dysthymia that are expected to last or have already lasted 6 months or more?”. We also considered including illicit drug interference, a proxy for substance use disorders, as a potential confounder. This derived variable assesses whether illicit drug use interfered significantly with usual routine, occupational functioning, or social relationships in the past 12 months (yes vs. no) [24]. However, this variable is subject to substantial underreporting, and therefore, was not considered.

### 2.5. Statistical Analysis

Cross-tabulations were performed initially examining the distributions of observations by suicidal ideation status for each covariate. Independent variables were screened for inclusion based on their univariate associations with suicidal ideation. Predictors that were significant at a liberal *p* value (*p*-value ≤ 0.2) were retained as candidates for the multivariable model. Three models were constructed using manual forward selection procedure with the socio-demographic variables (age, gender, household income) and clinical profile variables (chronic physical illness and mood disorder) were sequentially added to the model. To be more specific, model 1 assessed the unadjusted association between immigration identity and lifetime suicidal ideation, while model 2 adjusted for the effect of socio-demographic variables and model 3 adjusted for both socio-demographics and clinical profile variables.

For the multivariable logistic regression, if *p*-value for a predictor is less than 0.05, the predictor will be retained in the model. Age and gender are considered to be important confounders, which were adjusted in the model irrespective of their *p*-values. All statistical analyses were performed using SURVEYLOGISTIC procedure in SAS V.9.4 (SAS Institute Inc., Cary, NC, USA) adjusting for the complex survey sampling design. Among all participants, suicidal ideation was missing for 4.45%. All predictor variables had <5% missing data.

To assess whether a predictor has a confounding effect, a change of 20% or more in the coefficients of immigration identity was used as a cut-off [25]. Effect modifications were investigated by examining all possible two-way interactions for the main exposure variable with predictors and confounders included in the main effect model. Interactions were assessed based on their *p*-values and Akaike information criterion (AIC) comparisons. To examine model goodness of fit and predictability, receiver operating characteristic (ROC) curve was generated and area under the curve (AUC) was reported. The final results are presented as adjusted odds ratios (aOR) with 95% confidence intervals. 

## 3. Results

Of the 12,686 Canadian populants aged 15 years or more, 12,121 responded to the question on suicidal ideation, 1235 (10.19%) reported lifetime suicidal ideation, and 1858 (15.48%) were immigrants. Of the immigrations, 84.66% lived for 10 or more years in Canada (established immigrants) and 15.34% lived for 9 or less years (recent immigrants). Characteristics of study subjects stratified by the reported lifetime suicidal ideation are displayed in Table 1. 

In the univariate analysis (Table 2), immigrants were less likely to report suicidal ideation; whereas, among the immigrants, those who lived longer than 10 years were more likely to report suicidal ideation compared to the recent immigrants, who lived less than 10 years. Younger age, females, those who reported chronic physical conditions and mood disorders, were more likely to report suicidal ideation in the unadjusted analysis. There was no significant difference in reported suicidal ideation among participants with different level of education.

**Figure 1:**
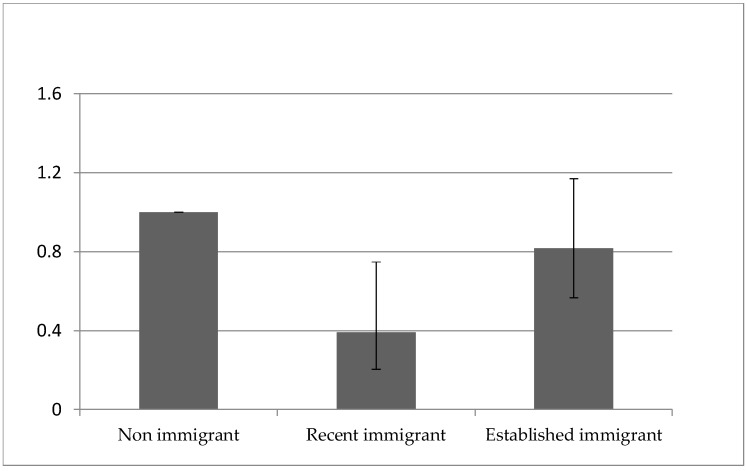
Adjusted odds ratio and 95% confidence intervals of self-reported suicidal ideation for recent and established immigrants compared to non-immigrants.

In the multivariable analyses (Table 3), the results based on model 2 indicated that the association between immigrant status and suicidal ideation remained significant after adjusting for socio-demographic variables, regardless of the duration of residence in Canada (recent immigrants: aOR = 0.26, 95% CI, 0.14–0.5, and established immigrants: aOR = 0.68, 95% CI, 0.48–0.96). The results of model 3 after adjusting for the socio-demographics and clinical profile variables indicated that the risk of suicidal ideation among recent immigrants remained significantly lower than Canadian-born population (aOR = 0.39, 95% CI, 0.20–0.74, *p*-value = 0.0044). However, for established immigrants, that health advantage disappeared after controlling for the effects of mood disorders and chronic illnesses (aOR = 0.82, 95% CI, 0.57–1.17, *p*-value = 0.2673). The increased risk of suicidal ideation against the length of residence in Canada in comparison with non-immigrants is further displayed in Figure 1. Model 3 achieved the highest area under the curve (AUC) of the receiver operating characteristic (ROC) of 0.734, compared to model 2 without clinical profile variables (AUC of 0.614) and model 1 without socio-demographic and clinical profile variables (AUC of 0.515). The Akaike information criterion (AIC) was also used to select the preferred model, which was model 3, with the lowest AIC (model 1: 3,094,232.5; model 2: 2,998,775.3; and model 3: 2,626,481.3). Therefore, model 3 is selected as the final model. 

The results of model 3 shows that younger age increases the odds of suicidal ideation being reported. Individuals aged less than 25 years old (aOR = 2.67, 95% CI, 1.81–3.94), and aged 25–40 years old (aOR = 2.01, 95% CI, 1.36–2.97), and aged 40 to 65 years old (aOR = 1.53, 95% CI, 1.11–2.10) were more likely to have suicidal ideation, when compared to individuals aged 65 or older. A similar trend was found with household income. Subjects who had low income had higher odds of reporting suicidal ideation than subjects who have middle or high income. The odds of subjects with household income less than $20,000 reporting suicidal ideation (aOR = 2.11, 95% CI, 1.45–3.08) were more than twice the odds of subjects with $80,000 of income. The odds of a female reporting suicidal ideation were more than 4% to 64% higher than the odds of a male, adjusting for age and immigration identity. However, after adjusting for mood disorders and chronic illnesses, differences across genders were not statistically significant (aOR = 1.14, 95% CI, 0.90–1.44, *p*-value = 0.2945). Consistent with the unadjusted results, diagnosed mood disorders were the strongest predictor of suicidal ideations in our model. Those who reported being diagnosed with mood disorders were close to ten times more likely to experience suicidal ideations compared to those who had no mental disorders (aOR = 9.7, 95% CI, 7.3–12.9).

**Figure 2:**
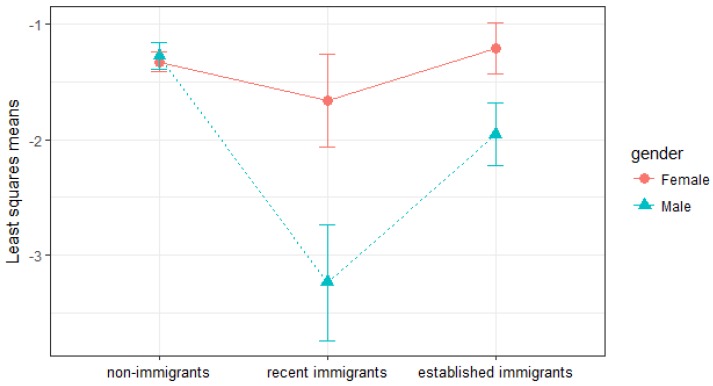
Least squares means (estimates of the linear predictors on the logit scale) and the 95% confidence intervals of suicidal ideation for non-immigrants, recent immigrants, and established immigrants, stratified by gender.

A significant interaction between gender and immigration identity (*p*-value = 0.0059) emerged when we evaluated all possible two-way interactions with the main exposure (*p*-values for interactions of the main exposure and age, income, mood disorders, and chronic medical illnesses were 0.995, 0.5249, 0.6070, and 0.7439, respectively). Figure 2 indicated female immigrants did not benefit from the protective effect of migration, regardless of the duration of residence in Canada after immigration. Compared to Canadian-born females, recent and established immigrant women have similar odds of reporting suicidal ideation (recent: aOR = 0.72, 95% CI, 0.32–1.59, established: aOR = 1.12, 95% CI, 0.71–1.78) (Table 4). Whereas immigrant males were at significantly lower risk than their Canadian-born counterparts (recent: aOR = 0.14 (95% CI, 0.05–0.39), *p*-value = 0.0001; established: aOR = 0.51, 95% CI, 0.29–0.89, *p*-value = 0.0168). The likelihood of suicidal ideation was similar among non-immigrant males and females. However, among immigrants, females were more likely to have suicidal ideation than males, and the likelihood increased as the duration of residence in Canada increases (Figure 2). 

## 4. Discussion

The results of this study add valuable, recent, and previously unavailable information on the association between suicidal ideation and immigration identity in Canada. It also deepens our understanding for the healthy immigrant effect and challenges the commonly accepted view that immigrants represent a homogeneous group. Although our data show that immigration identity is associated with a significantly lower risk of suicidal ideation when controlling for age, gender, and income, further adjustment clearly showed that most of that association is explained by the short duration of residence in Canada after immigration. When controlling for mood disorders and chronic illnesses, differences in reporting suicidal ideation among established immigrants and Canadian-born subjects are vanished. Gender influenced the likelihood of reporting suicidal ideation to a significant degree, in both recent and established immigrants. Females were significantly underprivileged and persistently had greater risk for suicidal ideation relative to immigrant males, irrespective to the extent of their stay in Canada after immigration.

The healthy immigrant effect observed in this study is not surprising. Previous studies have shown that Canadian immigrants are less likely to report symptoms related to depression [18], anxiety [19], and psychological distress [17]. One potential explanation for such an effect could be related to the positive selection process, whether due to self-selection or selection by Canada’s immigration policy [20]. Self-selection is based on a theory that immigrants are those who believe that better opportunities exist outside their native countries, and are able to endure the cost to experience these opportunities [20]. Thus, those who choose to immigrate would have better qualifications that allow them to believe they have better opportunities. Therefore, they could be systematically different from those who chose not to migrate. On the other hand, another important contributing factor would be the policies set for immigration by the government of Canada in order to control the size and composition of the stream of immigrants. Such policies aim to improve the human capital of the country and decrease health care costs [26]. Then, only those who are physically, educationally, and financially successful would have higher chances to be approved for immigration. Since these factors have also been linked to better health profile, consequently, positive selection can explain the better health profile exhibited by Canadian immigrants and their lower risk for reporting suicidal ideations.

Another significant finding from our analysis is the effect of duration (time since immigration). We observed a dissipation of the healthy immigrant effect over time for established immigrants, as they were not different from non-immigrants in term of reporting suicidal ideations. This finding goes in agreement with other studies assessing self-rated health, which described similar patterns [27]. On the other hand, a study assessing depression and alcohol dependence among Canadian immigrants reported sustained health benefits even after twenty years of residence in Canada [18]. To understand these changes, one needs to consider the challenges that face immigrants, which may include both cultural and structural factors. The effect of culture, commonly referred to as acculturation, is based on a theory that when immigrants abandon cultural characteristics apparently associated with their home country and adopt those of the receiving country, their health changes for the worse [28]. This process could be associated with stress due to factors related to assimilation and social integration within the host country. In 1986, Trovato reported that levels of social integration and assimilation were inversely related to suicide death among Canadian immigrants [29]. Acculturative stress has also been linked to suicidal ideation. A study assessing depression and suicidal ideations among Mexican immigrants in USA showed that those who had higher level of acculturative stress were at higher risk for reporting depression and suicidal ideation [30]. Another component, that has been suggested, but not thoroughly examined in the literature, is the effect of social structure, which may include unemployment, mismatched education and employment, and racial discrimination [31]. In time, the interplay between structural and cultural factors can create social inequalities, negatively impacting immigrants’ mental health, and might increase their risk of experiencing suicidal ideation. Another line of reasoning for the effect of duration can go with the possibility of a confounding cohort effect with immigration year. Factors related to home or host countries (after immigration) can specifically impact certain cohorts of immigrants and not others. A previous study examined that possibility and showed a significant cohort effect for self-rated heath and chronic illnesses [32].

In our analysis, there was a significant indication of an upturn in suicidal ideation risk in immigrant females. Usually, females are more likely to report suicidal ideation compared to males [7]. In general, gender is a strong determinant of mental health, and usually interacts with other determinants of health [33]. In the case of immigrant women, the change in sex roles and the rise in responsibilities at home and work can negatively impact not only their mental health, but also their access to mental health services [23]. They may experience disadvantaged social positions due to gender discrimination and victimization [34,35]. These factors could be more common or have a stronger impact among immigrant women. Women are usually considered at inferior position to men, therefore, they have lower power, status, and access to resources. Immigrant women experience greater gender discrimination as they work in different sectors than men, and occupy lower professional ranks and lower wage jobs [35], all of which would render them more susceptible to experience a worse mental health profile than their male counterparts.

Strengths of this study include the use of a complex survey design that allows for (1) generalizability of our findings to the Canadian non-institutionalized population aged 15 years or more; and (2) as study subjects were not recruited based on their immigration status, the potential for selection bias is minimal, as subjects participating in the survey are not systematically different from those who did not participate in terms of immigration status. However, the interpretation of our findings is limited by several factors. The most prominent of them is the cross-sectional design, which restricts evaluation of temporality, therefore, we cannot assume a causal association between immigration and suicidal ideation. Second, the CCHS data are based on self-reports, so it may be subject to recall bias and misinterpretation of survey questions. Third, subjects with suicidal ideations may choose not to participate in the survey due to their condition, which in turn affects the prevalence. Fourth, we could not eliminate the possibility of a confounding cohort effect with time of immigration where factors related to home or host countries can differentially affect particular cohorts of immigrants. Besides, we could not examine immigrant subgroups based on country of origin or ethnicity. Finally, CCHS results are limited to the Canadian civilian non-institutionalized population. The institutionalized population, as well as the homeless, may be more likely to present suicidal ideations, as well as of adverse health conditions [36].

Future research is warranted to examine the effect of ethnic differences among immigrants, since different ethnicities and different cultures might have different beliefs about suicide that would impact either reporting suicidal ideations or even experiencing them in the first place. Longitudinal cohort studies are required to examine trends and trajectories of mental illnesses among immigrants and their descendants, to further understand the effect of duration on the healthy immigrant effect in the context of suicidal behavior. Policymakers, practitioners, and mental health promotion initiatives will need to pay more attention to the heterogeneity across different immigrant subgroups, especially immigrant women, and established immigrants when considering suicidal behavior and mental health. There is also a need to consider public health interventions that support these groups and work towards decreasing their stress. 

## 5. Conclusions

Canadian immigrant health advantages extend to suicidal ideation, however, this effect was only temporary. Disparities among immigrant subgroups need to be carefully considered when planning future interventions and health promotion initiatives, especially for established immigrants (>10 years) and immigrant females. Future studies need to consider other important factors and include analyses of longitudinal data to further elucidate intersectional aspects of the “healthy immigrant effect” for suicidal ideation in Canada.

## Figures and Tables

**Table 1:** Sample characteristics by lifetime suicidal ideation.

Variable	Categories	Total (%)	Suicidal Ideation	
			Yes, *n* (%)	No, *n* (%)
Lifetime suicidal ideation		12,121	123 (10.19)	10,886 (89.91)
Immigrant identity (*n* = 12,002)	Yes	1858 (15.48)	157 (1.31)	1701 (8.92)
	No	10,144 (84.52)	1070 (14.17)	9074 (75.6)
Time since immigration (*n* = 1858)	0 to 9 years	285 (15.34)	18 (0.97)	267 (14.37)
	10 or more years	1573 (84.66)	139 (7.48)	1434 (77.18)
Combined immigration variable (*n* = 12,002)	Non-immigrant	10,144 (84.51)	1070 (8.91)	9074 (75.6)
	0 to 9 years	285 (2.37)	18 (0.15)	267 (2.22)
	10 or more years	1573 (13.11)	139 (1.16)	1434 (11.95)
Age (*n* = 12,121)	Youth	1240 (10.23)	162 (1.34)	1078 (8.89)
	Young Adult	1980 (20.17)	221 (1.82)	1759 (14.51)
	Middle adult	4300 (33.89)	518 (4.27)	3782 (31.2)
	Senior	4601 (31.7)	334 (2.76)	4267 (35.2)
Gender (*n* = 12,121)	Male	5293 (43.67)	480 (3.96)	4813 (39.71)
	Female	6828 (56.33)	755 (6.23)	6073 (50.1)
Household income (*n* = 12,101)	<$20,000	1364 (11.27)	247 (2.04)	1117 (9.23)
	$20,000–$39,999	2773 (22.92)	301 (2.49)	2472 (20.43)
	$40,000–$59,999	2331 (19.26)	222 (1.83)	2109 (17.43)
	$60,000–$79,999	1777 (14.68)	161 (1.33)	1616 (13.35)
	>$80,000	3856 (31.87)	300 (2.48)	3556 (29.39)
Level of education (*n* = 11,954)	<Secondary school	2316 (19.38)	221 (1.85)	2095 (17.53)
	Secondary school	2603 (21.78)	294 (2.46)	2309 (19.32)
	Some post-secondary	536 (4.48)	79 (0.66)	457 (3.82)
	Post-secondary certificate	6499 (54.37)	623 (5.21)	5876 (49.16)
Chronic health conditions (*n* = 12,121)	Yes	7701 (63.53)	919 (7.58)	6782 (55.95)
	No	4566 (37.67)	316 (2.61)	4104 (33.86)
Diagnosed with mood disorder (*n* = 12,104)	Yes	1090 (8.12)	488 (4.03)	602 (4.97)
	No	11,014 (91)	743 (6.14)	10,271 (84.86)

**Table 2:** Results of univariate analysis of lifetime suicidal ideation.

Variable	Category	Odds Ratio	95% CI		*p*-Value
			Upper	Lower	
Immigrant (Ref: non-immigrant)	Yes	0.57	0.47	0.78	0.0004
Time since immigration (Ref: 0 to 9 years)	10 or more years	2.04	1.00	4.17	0.0495
Immigration identity (Ref: non- immigrant)	0 to 9 years	0.32	0.17	0.62	0.0003
	10 or more years	0.66	0.47	0.93	
Age (Ref: senior)	Youth	1.67	1.21	2.29	0.0133
	Young adult	1.39	0.99	1.93	
	Middle adult	1.33	1.02	1.72	
Gender (Ref: male)	Female	1.35	1.07	1.69	0.0102
Household income (Ref: <$20,000)	$20,000–$39,999	0.66	0.47	0.93	<0.0001
	$40,000–$59,999	0.38	0.27	0.52	
	$60,000–$79,999	0.40	0.28	0.57	
	>$80,000	0.399	0.29	0.56	
Level of education (Ref: <secondary school)	Secondary school	0.85	0.64	1.12	0.1809
	Some post-secondary	1.05	0.77	1.42	
	Post-secondary certificate	1.28	0.79	2.07	
Chronic health conditions (Ref: no)	Yes	1.83	1.47	2.41	<0.0001
Diagnosed with mood disorder (Ref: no)	Yes	11.04	8.47	14.38	<0.0001

**Table 3:** Multivariable logistic regression models to assess the association of immigration identity and suicidal ideation adjusting for various confounders (*n* = 11,967).

		Model 1	Model 2	Model 3	*p*-Value ^1^
Immigrant (Ref: no)	9 or less years	0.32 (0.17–0.62)	0.26 (0.14–0.50)	0.39 (0.20–0.75)	0.0044
	10 or more years	0.66 (0.47–0.93)	0.68 (0.48–0.96)	0.82 (0.57–1.17)	0.2673
Age (Ref: senior)	Youth		1.88 (1.31–2.68)	2.67 (1.81–3.94)	<0.0001
	Young adult		1.85 (1.29–2.66)	2.01 (1.36–2.97)	0.0005
	Middle adult		1.56 (1.17–2.07)	1.53 (1.11–2.10)	0.0092
Gender (Ref: male)	Female		1.31 (1.04–1.64)	1.14 (0.90–1.44)	0.2945
Household income (Ref: >80,000)	<$20,000		2.97 (2.09–4.21)	2.11 (1.45–3.08)	0.0001
	$20,000–$39,999		2.00 (1.43–2.81)	1.695 (1.18–2.43)	0.0042
	$40,000–$59,999		1.06 (0.78–1.43)	0.93 (0.68–1.27)	0.6399
	$60,000–$79,999		1.09 (0.78–1.53)	1.00 (0.69–1.44)	0.9946
Chronic health conditions (Ref: no)	Yes			1.77 (1.36–2.28)	<0.0001
Diagnosed with mood disorder (Ref: no)	Yes			9.7 (7.3–12.9)	<0.0001

Model 1: Unadjusted crude model; Model 2: Adjusted for age, gender, household income; Model 3: Adjusted for the variables included in model 2 as well as chronic health conditions and mood disorders. ^1^ *p*-values for variables included in model 3.

**Table 4:** Adjusted odds ratio (aOR) and 95% confidence intervals of suicidal ideation for recent and established immigrants vs. non-immigrants stratified by gender.

	aOR	95% CI		*p*-Value
		Lower	Upper	
Females				0.6176
Non-immigrant	1.00	-	-	-
Recent immigrant	0.72	0.32	1.59	0.4118
Established immigrant	1.12	0.71	1.78	0.6179
Males				<0.0001
Non-immigrant	1.00	-	-	-
Recent immigrant	0.14	0.05	0.39	0.0001
Established immigrant	0.51	0.29	0.89	0.0168
